# A Caustic Concern: A Case of a Chemical Burn

**DOI:** 10.7759/cureus.90592

**Published:** 2025-08-20

**Authors:** Anais Panossian, Justin Peterson

**Affiliations:** 1 Department of Medicine, University of California Los Angeles David Geffen School of Medicine, Los Angeles, USA

**Keywords:** acute burn, alkaline chemical burn, altered mental status, chemical exposure, rhabdomyolysis

## Abstract

Chemical burns are a less frequently encountered cause of dermatologic burns and can have varied presentations depending on the chemical, exposure, host factors, and post-exposure interventions. We present a case of a diffuse chemical burn from a basic irritant with potential concomitant other chemical exposures in a patient with altered mental status from polypharmacy. Prompt recognition of a chemical burn can be diagnostically challenging but is paramount for initiating appropriate treatment and mitigating adverse outcomes.

## Introduction

The skin is the body’s first natural defense and barrier against pathogens and assists in regulating heat and fluid loss. However, this barrier can be disrupted by several means, including, but not limited to, burns. Damage caused by burns is due to acute injury of the skin or subcutaneous tissue when it comes in contact with exposure to cold, heat, radiation, electricity, and/or chemical agents [1]. Chemical agents that are responsible for burns are generally categorized as acidic or basic, and their spectrum of presentation presents a diagnostic challenge. Although there are more than one million chemical compounds, the National Fire Protection Society has declared about 300 of them as severely hazardous substances to health, some of which are common and can be found in the household setting [2]. When distinguishing between the two types of chemical burns, i.e., acidic or basic chemicals, it is notable that acidic chemicals generally cause a shallower burn as they work by denaturing and coagulating proteins. On the other hand, chemicals with a basic pH tend to cause deeper burns by a process called saponification, which can result in necrosis of the lipid barrier in the surface epithelium and subcutaneous tissue of the skin, penetrating more of the deeper skin layers [3,4].

Chemical burns in general are responsible for about 10.7% of all burn injuries and about 30% of deaths caused by burns [5]. It is interesting to note that many of these caustic chemicals are found in many everyday household items or commonly bought substances in stores. Common alkali chemicals include bleach, oven cleaners, drain cleaners, and floor strippers. These chemicals can differ in their main chemical compounds. Floor strippers, for example, include the compound sodium hydroxide, while bleach has sodium hypochlorite. Both are alkali and, as a result, can cause severe burns by liquefactive necrosis and cleaving of the lipids and proteins in skin and subcutaneous tissue; however, when mixed with water, sodium hydroxide results in a strong exothermic reaction and thus releases heat [6,7]. It is imperative to recognize such chemical burns, as they often result in significant scarring, possible contractures, and blindness when involving the eyes. Immediate first aid management of removing clothing from the affected area and irrigating with copious cool water to dilute and run off the chemical is necessary to reduce further damage [8]. Here, we describe a case of an acute chemical burn caused by several chemicals, including floor stripper, a strong basic chemical.

## Case presentation

A 65-year-old male presented to the emergency department after being found unresponsive in his home. The patient was found in his home by his ex-girlfriend with an altered mental status. He reported to her by telephone earlier in the day that he was “dragging” himself around the apartment floor for hours and that he “took too many sleeping pills.” The patient was reportedly known to be well the day prior to presentation.

His past medical history was notable for an abdominal aortic aneurysm, obstructive sleep apnea, and a previous dengue fever infection. His current medications included paroxetine, quetiapine, bupropion, alprazolam, and amphetamine-dextroamphetamine. The patient was a former smoker, denied regular or heavy alcohol use, and denied using illicit drugs. He worked as an artist.

**Figure 1:**
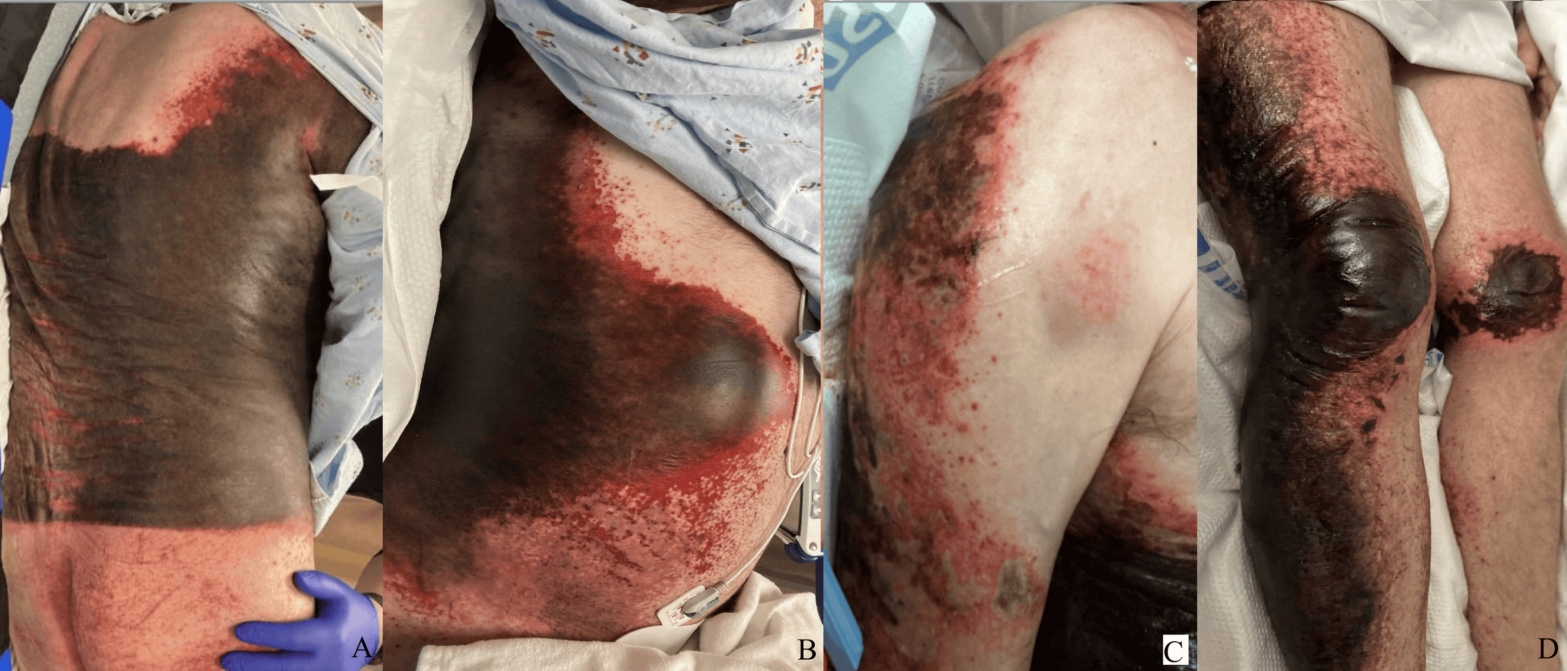
Examination results on presentation A. back, B. abdomen, C. right upper extremity, D. lower extremities

On arrival, the patient had a temperature of 100.0°F, heart rate of 116 beats per minute, respiratory rate of 20 breaths per second, blood pressure of 106/65 mmHg, and an oxygen saturation of 94%. On exam, he was noted to be restless in bed and mildly agitated. He was oriented to name but not to place or time. He was noted to be mildly tremulous without focal neurologic deficits. Cardiac exam was notable for regular tachycardia; pulmonary and abdominal exams were unremarkable. His skin exam revealed diffuse leathery black-to-red ecchymoses with purpuric plaques of the right torso (Figure 1a, 1b) up to the right shoulder (Figure 1c), extending down the right lower extremity and on the medial side of his left knee (Figure 1d). The rash had retiform change to geometric borders and adjacent scattered, somewhat punched-out papules. It had sharp demarcation lines, with the most notable being a well-demarcated linear scar across the lower lumbar back at the patient’s waistline. There was no gingival involvement. The rash was not tender or warm to the touch. The laboratory values are presented in Table 1.

**Table 1:** Laboratory values

Laboratory test	Result	Reference range
White blood cell count	11.35 ×10E3/uL	4.16-9.95 x10E3/uL
Hemoglobin	16.3 g/dL	11.6-15.2 g/dl
Platelets	229 ×10E3/uL	143-398 x10E3/uL
Sodium	140 mmol/L	135-146 mmol/L
Potassium	3.7 mmol/L	3.6-5.3 mmol/L
Bicarbonate	20 mmol/L	20-30 mmol/L
Blood urea nitrogen (BUN)	29 mg/dL	7-22 mg/dL
Creatinine	3.42 mg/dL	0.6-1.3 mg/dL
Aspartate aminotransferase (AST)	1116 U/L	13-62 U/L
Alanine aminotransferase (ALT)	298 U/L	8-70 U/L
Alkaline phosphatase	41 U/L	37-133 U/L
Total bilirubin	0.9 mg/dL	0.1-1.2 mg/dL
Calcium	8.9 mg/dL	8.6-10.4 mg/dL
Magnesium	1.8 mg/dL	1.4-1.9 mg/dL
Phosphorus	2.8 mg/dL	2.3-4.4 mg/dL
International normalized ratio (INR)	1.3	
Prothrombin time	15.7 seconds	11.5-14.4 seconds
Activated partial thromboplastin time	29.6 seconds	24.4-36.2 seconds
Fibrinogen	454 mg/dL	235-490 mg/dL
Creatine kinase	70.875 U/L	63-473 U/L
D-dimer	9.19 ug/mL	<0.60 ug/mL
Serum uric acid	12.2 mg/dL	3.4-8.8 mg/dL
Hepatitis A IgM antibody	Nonreactive	
Hepatitis B surface antigen	Nonreactive	
Hepatitis B surface antibody	117 mIU/mL	<8.5 mIU/mL
Hepatitis B core antibody	Nonreactive	
Hepatitis C antibody	Nonreactive	
Ethyl alcohol	<15 mg/dL	<15 mg/dL
Peripheral smear	Slight burr cells	
Blood cultures (two sets)	No growth	

The patient was started on intravenous hydration for rhabdomyolysis. His creatinine kinase levels and creatinine levels were trended and improved with intravenous hydration. Hematology was consulted and felt that disseminated intravascular coagulation (DIC) was unlikely given the relatively normal results of his coagulation studies. Dermatology was consulted, and suspected that the patient had an exogenous source for the rash, such as a chemical burn. They performed two punch biopsies of the skin in the right lower extremity and right upper extremity, which revealed transepidermal necrosis. Given the high suspicion of a chemical burn, the patient’s ex-girlfriend returned to the patient’s home. She discovered liquid on the floor where the patient was found with a knocked-over empty bottle of First Street Heavy Duty Floor Stripper, as well as nearby empty bottles of acetone, Goof Off (solvent remover), and artist’s paint thinner. The floor stripping fluid is a strong caustic base, and after discussion with dermatology and plastic surgery, this finding confirmed a diagnosis of a severe chemical burn estimated at 25% burn surface area (BSA). The basic floor stripper chemical solution was felt to be the primary driver of the patient's dermatologic injuries, with the other substances as potential contributors to weakening the patient's skin barrier and further promulgating tissue injury. The patient was transferred to a burn center to continue treatment for his chemical burn and eventually recovered and was discharged home. When his mental status improved, the patient confirmed that he had taken several sleeping pills on the day prior to presentation and that he had slipped and fallen at the refrigerator. He found that he subsequently could not stand up, so he dragged himself around the apartment for hours before he was discovered.

## Discussion

This case demonstrates an unusual and diagnostically challenging presentation of chemical burns caused by caustic household agents. The patient’s presentation initially raised concern for medical emergencies such as DIC, vasculitis, meningococcemia, toxic shock syndrome, other hemorrhagic fevers, and warfarin skin necrosis; however, physical exam, laboratory findings, imaging, and biopsy results combined with consultation from specialists pointed to the underlying cause of an exogenous chemical burn injury.

Chemical burns are an important consideration in the differential diagnosis of patients presenting with cutaneous lesions, especially ones that are atypical in morphology. In this case, the “sharply demarcated, retiform, and geometric distribution of the rash, as well as the spared mucosal surfaces,” were key physical exam findings suggesting external exposure. The spilled industrial-grade floor stripper - alongside acetone and paint thinner - further supported the suspicion of chemical injury. Based on the physical exam findings and the solutions present in the patient’s home, the burns of this patient were consistent with extreme alkali chemical compound burns. The patient’s prolonged immobility and confusion, combined with him being fully clothed, likely compounded the tissue injury, allowing the solution to be soaked into the fabric and be in close contact with the skin for a longer period. Extensive time-exposure leads to further tissue damage and injury, resulting in more severe burns as seen in this case.

There is a possibility of other chemicals, such as acetone, solvent remover, and paint thinner, being mixed with the solutions the patient was exposed to, which may not be as caustic as floor stripper, but can still cause skin irritation and inflammation after prolonged exposure and contribute to the damage that occurred. In addition, this patient’s at-home medication regimen of central nervous system depressant agents and stimulants could have contributed to his altered mental status and inability to avoid harm. The laboratory findings of significantly elevated creatine-kinase levels (70.875 U/L) and acute kidney injury also underscored the systemic consequences of prolonged muscle compression and potential rhabdomyolysis. The skin biopsy results of transepidermal necrosis were consistent with chemical burns but nonspecific and therefore highlight the importance of clinical context and collateral history in medical practice, which helped confirm the diagnosis in this case.

Ultimately, prompt recognition and transfer to a specialized burn center facilitated this patient’s recovery. Immediate management that would have been helpful to reduce permanent damage would be to remove clothing and irrigate with cool water. This case suggests the importance of clinicians remaining vigilant for environmental exposures in atypical dermatological presentations, particularly in vulnerable populations such as the elderly, patients with substance use disorder, or those with underlying psychiatric illnesses. 

## Conclusions

Chemical burns represent a minority but clinically significant number of burn cases. The clinical manifestations of chemical burns vary, but well-demarcated rashes or those confined to areas of clothing may represent a diagnostic clue of a potential chemical exposure. Removing saturated clothing and irrigating any ongoing exposures can help mitigate the extent of damage done to the patient. Identifying the type of chemical injury can help in diagnosis, treatment, and prognosis. In this case, an altered mental status secondary to polypharmacy contributed to extensive exposure to chemical irritants, which resulted in a more diffuse and severe chemical burn. 
